# Miniaturized supercapacitors: key materials and structures towards autonomous and sustainable devices and systems

**DOI:** 10.1016/j.jpowsour.2016.04.131

**Published:** 2016-09-15

**Authors:** Francesca Soavi, Luca Giacomo Bettini, Paolo Piseri, Paolo Milani, Carlo Santoro, Plamen Atanassov, Catia Arbizzani

**Affiliations:** a Department of Chemistry “Giacomo Ciamician”, Alma Mater Studiorum – Università di Bologna, Via Selmi, 2, 40126 Bologna, Italy; b CIMaINa and Physics Department, Università degli Studi di Milano, Via Celoria 16, 20133 Milano, Italy; c Department of Chemical & Biological Engineering, Center for Micro-Engineered Materials (CMEM), University of New Mexico, Albuquerque, NM 87131, USA

**Keywords:** Flexible micro supercapacitor, Electrochemical double layer, Supersonic cluster beam deposition, Electrolyte gated transistor, Microbial fuel cell

## Abstract

Supercapacitors (SCs) are playing a key role for the development of self-powered and self-sustaining integrated systems for different fields ranging from remote sensing, robotics and medical devices. SC miniaturization and integration into more complex systems that include energy harvesters and functional devices are valuable strategies that address system autonomy. Here, we discuss about novel SC fabrication and integration approaches. Specifically, we report about the results of interdisciplinary activities on the development of thin, flexible SCs by an additive technology based on Supersonic Cluster Beam Deposition (SCBD) to be implemented into supercapacitive electrolyte gated transistors and supercapacitive microbial fuel cells. Such systems integrate at materials level the specific functions of devices, like electric switch or energy harvesting with the reversible energy storage capability. These studies might open new frontiers for the development and application of new multifunction-energy storage elements.

## Highlights

• Novel supercapacitor integration approaches for multifunction autonomous devices.
• Flexible supercapacitive electrodes fabricated by Supersonic Cluster Beam Deposition.
• Strategies to miniaturize supercapacitive low-power electrolyte gated transistors.
• Supercapacitors, electrolyte gated transistors and microbial fuel cells integration.

## Graphical abstract

**Figure:**
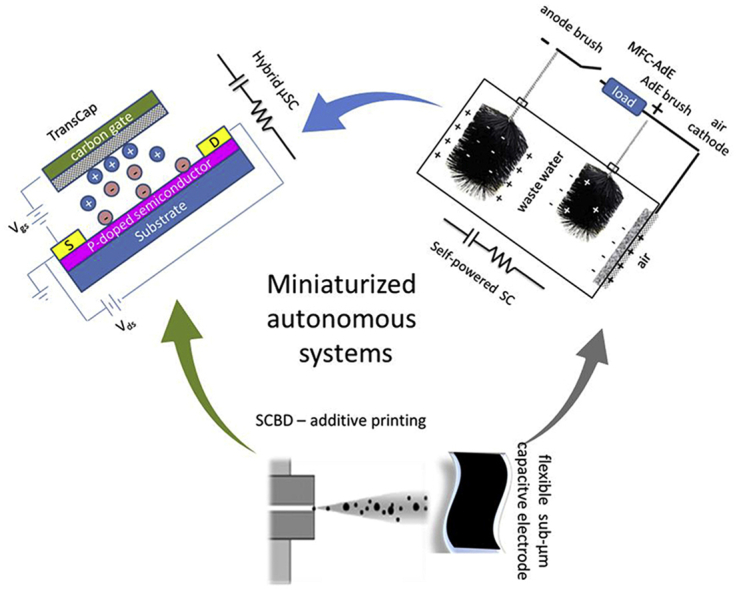

## 1 Introduction

Worldwide electric energy consumption from ICT and home entertainment consumer electronics is fast growing. While each device consumes a small quantity of electricity, power use in stand-by mode and massive widespread use make the cumulative consumption considerable. It has been estimated that the global energy demand of network-enabled devices is expected to reach around 1140 TW h by 2025 corresponding to an increase of 6% of current total global electricity consumption [1]. Efforts are required to power down or reduce and minimize energy requirements without losing functionality. On the other hand, energy autonomy is critical for remote sensing, robotics and medical applications like wearable and skin-attachable sensors [2] and implantable devices for in vivo diagnostics [3].

The design of autonomous systems includes a nanogenerator that harvests energy from the environment (photovoltaic, thermoelectrics, mechanical vibration, piezoelectric, bio- or enzymatic fuel cells), an energy storage unit (like a microbattery or a microsupercapacitor) that stores the harvested energy and delivers the power required to switch on function units like sensors and transceivers [2]. The key issue of autonomous systems is that energy harvesters typically provide power and voltage output that can be lower than what is required to charge conventional batteries and supercapacitors (hence for energy storage) and to switch on the function device. Hence, viable strategies are: i) the miniaturization of the energy storage system; ii) the decrease of power demand of the function device; and iii) the integration of different system components. On the other hand, the above reported applications have raised the problem of developing sufficiently compact and/or flexible, multifunctional electronic components by sustainable processes like printing methods [4], [5], [6], [7], [8], [9], [10], [11], [12], [13], [14], [15].

Within this context microsupercapacitors (μSCs) are playing a key role. μSCs are high power systems that may outperform μ-batteries in applications having high peak-to-average power demand due to their capability to store/deliver charge in relatively short times [12]. Compared to μ-batteries, μSCs feature much lower energy density but exhibit superior cycling stability, which is of paramount importance for applications (e.g. sensors) where repeated power peaks are required. Several classes of supercapacitors making use of electrodes with different nature and composition have been demonstrated [16], [17]. The most common are the electrochemical double layer capacitors (EDLCs) that use high surface area carbon electrodes which store/deliver charge by an electrostatic process. Pseudo-supercapacitors feature battery-like electrodes (electronically conducting polymers and metal oxides) that are charged/discharged by fast and reversible redox processes. Hybrid supercapacitors feature positive and negative electrode materials of different nature that are charged/discharged via different electrostatic and faradic modes. Carbons and polymers are key materials for μSCs since they are easily processable for in-plane or flexible architectures.

A plethora of electrode and electrolyte combinations have been proposed to yield supercapacitors with operating voltages varying from 1 V (aqueous systems) to 3 V and above (organic electrolytes, ionic liquids) and specific energy and power values spanning the 5–40 W h kg^−1^ and 1–6 kW kg^−1^ ranges [17]. This high “electrical response flexibility” of μSCs is crucial for their implementation into autonomous devices. Specifically, carbonaceous materials can store charge within a wide working voltage range which is limited only by the electrochemical stability of the electrolyte that for aqueous electrolyte is constrained below 2 V. For this reason, EDLCs can be successfully combined with a wide range of energy harvesters including those operating at low voltage like photovoltaics (PVs) [18] or enzymatic and microbial fuel cells [19].

Furthermore, a valuable, key aspect of supercapacitor electrodes is that many materials exhibit strategic additional properties that can be exploited for specific applications, along with their capability of reversibly storing charge and, thus, energy. This enables integration of the energy storage capability and of the target function at materials level [20]. Pseudocapacitive polymers, like poly(pyrrole), poly(3,4ethylenedioxythiophene), poly(3-alkylthiophenes) and poly(aniline), have specific mechanical, optical and electronic properties that are exploited in actuators, electrochromic devices, and sensors [21], [22]. Metal oxides are also used as mediators for electrocatalytic processes (e.g. MnO_2_ and RuO_2_) and may feature electrochromic and semiconductive properties (e.g.WO_3_ and TiO_2_) [23]. The semiconductivity of polymers and inorganic compounds is exploited in transistors. These widely used circuital electronic components are at the basis of several functionalities, from sensors to memories. In particular, electrolyte gated transistors (EGTs) are attractive candidates to be coupled to energy harvester/storage microsystems for their low-voltage operation characteristics [24]. In EGTs, the charge carrier density in the semiconductive channel can be reversibly modulated upon application of a bias (V_gs_) between the channel and a gate electrode through an electrolyte. Current (I_ds_) flows through the channel by application of a voltage (V_ds_) between drain and source (Fig. S1). Coupling pseudocapacitive channels like polymers and metal oxides with high double-layer capacitance carbon gates enables current modulations of several orders of magnitude at relatively low V_gs_ which positively affects power consumption and device stability (Fig. S1).

**Fig. 1:**
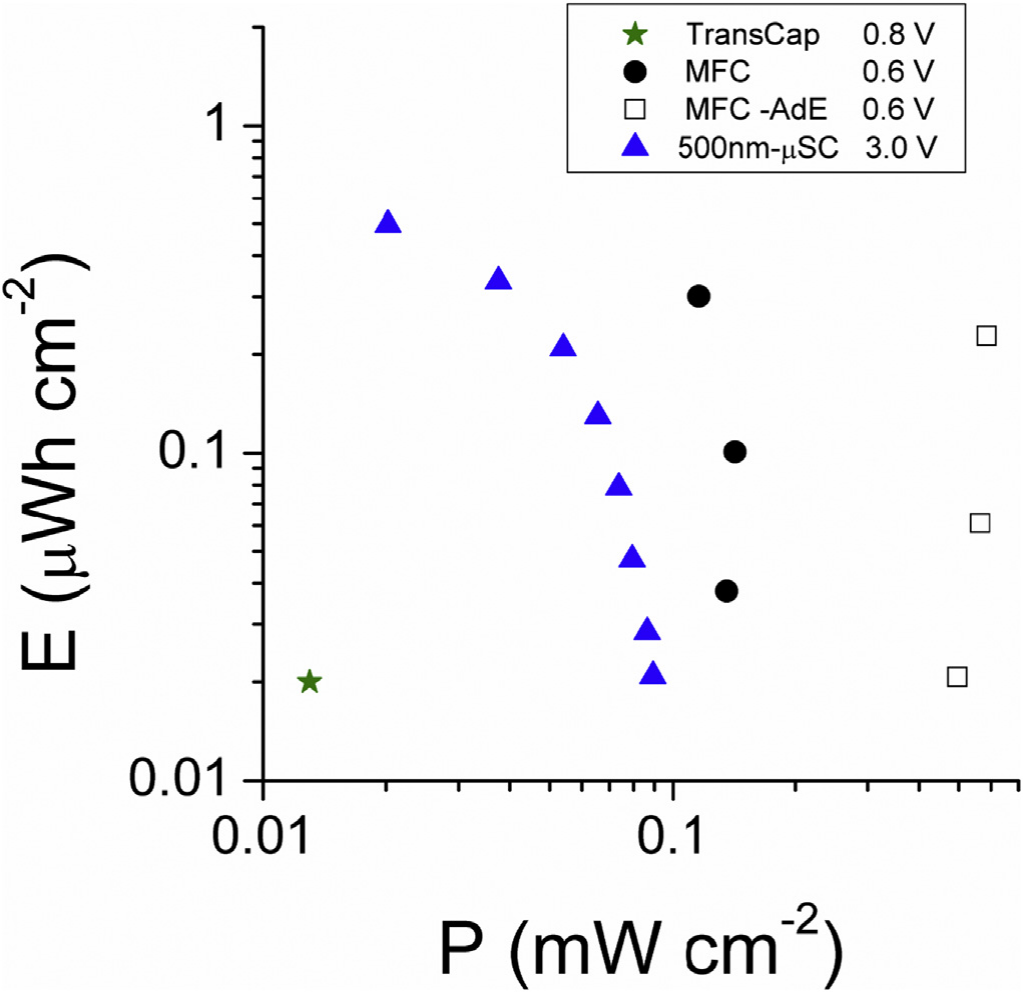
Ragone plots at RT of a TransCap with 50 nm-MEHPPV channel and N_1113_TFSI electrolyte with Vgs of 0.8 V at 28 μA cm^−2^; of the MFC (1.3–2 mA cm^−2^) and MFC-AdE (3.3–8.3 mA cm^−2^) cells with AC cathodes (0.6 V OCV), of a flexible μSC with 500 nm ns-C electrodes deposited on Mylar by SCBD and N_1113_TFSI electrolyte (20–180 μA cm^−2^, 3 V).

Sub-1V operation of EGTs has been demonstrated by combining activated carbon gates with polymer or inorganic channels and with both aqueous and organic electrolytes [25], [26], [27], [28], [29]. Specifically, p-type EGTs with poly(3,4-ethylenedioxythiophene) doped with poly(styrene sulfonate) (PEDOT:PSS) and poly[2-methoxy-5-(2′-ethylhexyloxy)-1,4-phenylene vinylene (MEH-PPV) p-type polymer channels were considered for their great potential for flexible, low-cost and easily processable electronic devices. PEDOT:PSS is one of the most investigated materials for Organic EGTs (OECTs) to be used in bioelectronic implants, chemical and biological sensors, and lab-on-a-chip systems [26], [30]. MEH-PPV is well known for its electroluminescence and for its potential applications to optical and electronic devices such as light-emitting diodes (LEDs) [31]. As it concerns inorganic channels, WO_3_ is well investigated for its application in electrochromism, sensing, photocatalysis and photoelectrochemistry [32]. The electric response of the organic and inorganic EGTs making use of high surface area carbon gate and IL-based electrolytes are summarized in Table S1. Table S1 also reports the energy required to drive EGTs (E_EGT_) that includes the contributions to dope the channel (E_gs_ = |V_gs_ Q_ch_|) and to let the current flowing through the channel (E_ds_) over a certain time (t). E_ds_ depends on the power dissipated through the channel (P_ds_ = |I_ds_ V_ds_|) (see SI). The doping charges and V_gs_ are in the 100–300 μC and 0.8–1.2 V ranges and the E_gs_ are of 0.03–0.1 μW h. The channel/electrolyte/gate stacking of EGTs can be viewed as a 2-electrode electrochemical cell where the capacitive channel and carbon gate components reversibly store charge at given V_gs_. Specifically, the channel/electrolyte/carbon gate stacking is analogous to that of a hybrid μSC where channel and gate electrodes are charged/discharged by a faradic and an electrostatic process, respectively. This means that the E_gs_ energy used to dope the channel is stored and, then, deliverable upon the EGT switch OFF. For the different EGTs here considered up to 50% of the energy spent to drive the EGT can be stored (E_stored_ = 100 Egs/E_EGT_), and thus, saved (Table S1). More importantly, these results point to the integration of EGT and μSCs at materials level, which has been demonstrated with TransCap [27]. TransCap featured an MEHPPV channel, a gate based on commercial activated carbon layer deposited on carbon paper and the ionic liquid N_1113_TFSI. The device could simultaneously work as EGT and μSC. The E_gs_ stored during the potentiostatic (PS) switch on at 0.8 V was ca 0.02 μWh and it was delivered during the following switch OFF (0 V) with an efficiency of 99.5% (Fig. S1) [27]. The energy (E) and power (P) delivered under a conventional galvanostatic discharge at 10 μA cm^−2^ (normalized to the electrode area) where 0.02 μW h cm^−2^ and 13 μW cm^−2^. These energy and power performance of TransCap are reported in the Ragone plot of Fig. 1. E (in Wh cm^−2^) and P were calculated by the integral of the cell voltage over time by using the eqs. (1), (2):

(1) E=i∫Vⅆt/3600

(2) P=E/Δt,

where Δt is the discharge time.

EGTs featuring carbon gates and TransCap represent a novel approach for the exploitation of the capacitive feature of electrodes into multifunction electrochemical devices.

High surface area carbons are also widely investigated as catalysts or catalyst supports for various types of fuel cells [33], [34]. Particularly innovative are microbial fuel cells (MFC), bioelectrochemical systems specifically devoted to convert organic compounds into electricity.

MFCs have been widely and successfully combined with external supercapacitors (MFC/SC) to provide suitable power output for switching on devices [19], [35], [36], [37], [38], [39]. The voltage of a single MFC generally does not exceed 0.7 V and therefore the external supercapacitors are also coupled with voltage amplifiers to enlarge the possible utilization of the discharge pulses [19]. The harvesting of energy output from benthic MFC for sensors applications has also been explored [40], [41]. The main disadvantage related with this MFC/SC coupling is the long time for recharging the outer SCs. While EDLCs featuring high capacitance (∼Farad) can store/deliver a high amount of energy harvested by the MFCs, they need long time to be recharged at the low currents generated by MFCs (typically ≪ 1 mA). This may limit the frequency at which SC discharge pulses, which are required to switch on function devices like sensors, are repeated. From literature achievements, the recharging time of MFC/SC systems varies within several minutes up to hours making the discharge and consequent utilization of high quality energy discontinuous with long standby periods [19].

The MFC electrodes are based on conductive materials with high surface area that coincide with the needs of supercapacitors. The natural presence of ions into the operating solution also is desirable for the counter ions migration towards the polarized electrode. These features have been exploited to develop an innovative concept of supercapacitive MFC in which the anode and the cathode of a single chamber membraneless MFC were used as negative and positive electrode of an internal SC which is self-recharged by the MFC redox reactions [42]. In a working MFC, electrogenic microorganisms colonize the anode electrode and oxidize the available substrate (i.e. the electron donor). The anodes feature high surface area and conductive carbonaceous 3D materials in order to accommodate electroactive biofilm and enhance the electrode/bacteria interaction [43] and their capacitive features have been already investigated in aqueous media [44]. On the other hand, MFC air-breathing cathodes are typically prepared with carbon porous supports and components that are also investigated for SCs (activated carbons, mesoporous carbons, etc). In rest, the bio-anode and oxygen cathode redox couples move the electrodes at potentials that are at more negative and positive, respectively, than the typical value exhibited by not polarized carbons in de-aerated aqueous electrolytes (near 0 mV vs Ag/AgCl [16]). This causes electrode polarization along with the formation of electrochemical double layers (EDLs) at the electrode interfaces without the application of any external voltage bias. Therefore, an internal, charged SC is formed and anode and cathode of the MFC can be directly used as the negative and positive electrodes [42]. The cell can be electrostatically and rapidly discharged; during the subsequent rest period the equilibrium electrode potentials are restored, the surface of carbon electrodes is polarized again and, therefore, the internal SC is recharged (Fig. S2). Figure S2b shows the cell voltage and electrode potential profiles of an MFC with a brush anode and an activated carbon (AC) based cathode at 2 mA cm^−2^ (i, normalized to the cathode area). This current density is 5 time higher than the value (0.6 mA cm^−2^) where maximum instant power P = iV (≈110 μW cm^−2^) is achievable under conventional MFC operation [45]. The cell capacitance (slope of the discharge capacity vs. voltage) is 12 mF cm^−2^ and the delivered E and P (eqs. (1), (2))) are 8.8 μW h cm^−2^ and 180 μW cm^−2^, not negligible values of interest for powering small electronic components like sensors. This performance is seriously affected by the high cell ohmic drop to which mainly contributes the air-breathing cathode. A 4 time decrease of the cathode ohmic drop was achieved by the use of an additional capacitive electrode (AdE) that was short-circuited with the cathode [42]. This approach positively affected E and P. Figure S2c shows the cell voltage trend during two discharges at 4 mA cm^−2^ followed by a rest period of the cell with AdE (MFC-AdE). Notably, during rest, MFC-AdE recovered the cell voltage value exhibited before discharge within 20 s, thus demonstrating the fast self-rechargeability of the SC. Fig. 1 reports the energy and power performance of MFC and MFC-AdE tested at 1.3–2 mA cm^−2^ and 3.3–8.3 mA cm^−2^. The highest values of E and P were 0.3 μW h cm^−2^ and 0.14 mW cm^−2^, and 0.4 μW h cm^−2^ and 0.6 mW cm^−2^ respectively. The comparison of these values with those reported in Table S1 and Fig. 1 demonstrates that the energy harvested by supercapacitive MFCs and, mostly MFC-AdE, can be delivered at power levels that are sufficiently high to drive electronic components like the Sub-1V EGTs and TransCap.

**Fig. 2:**
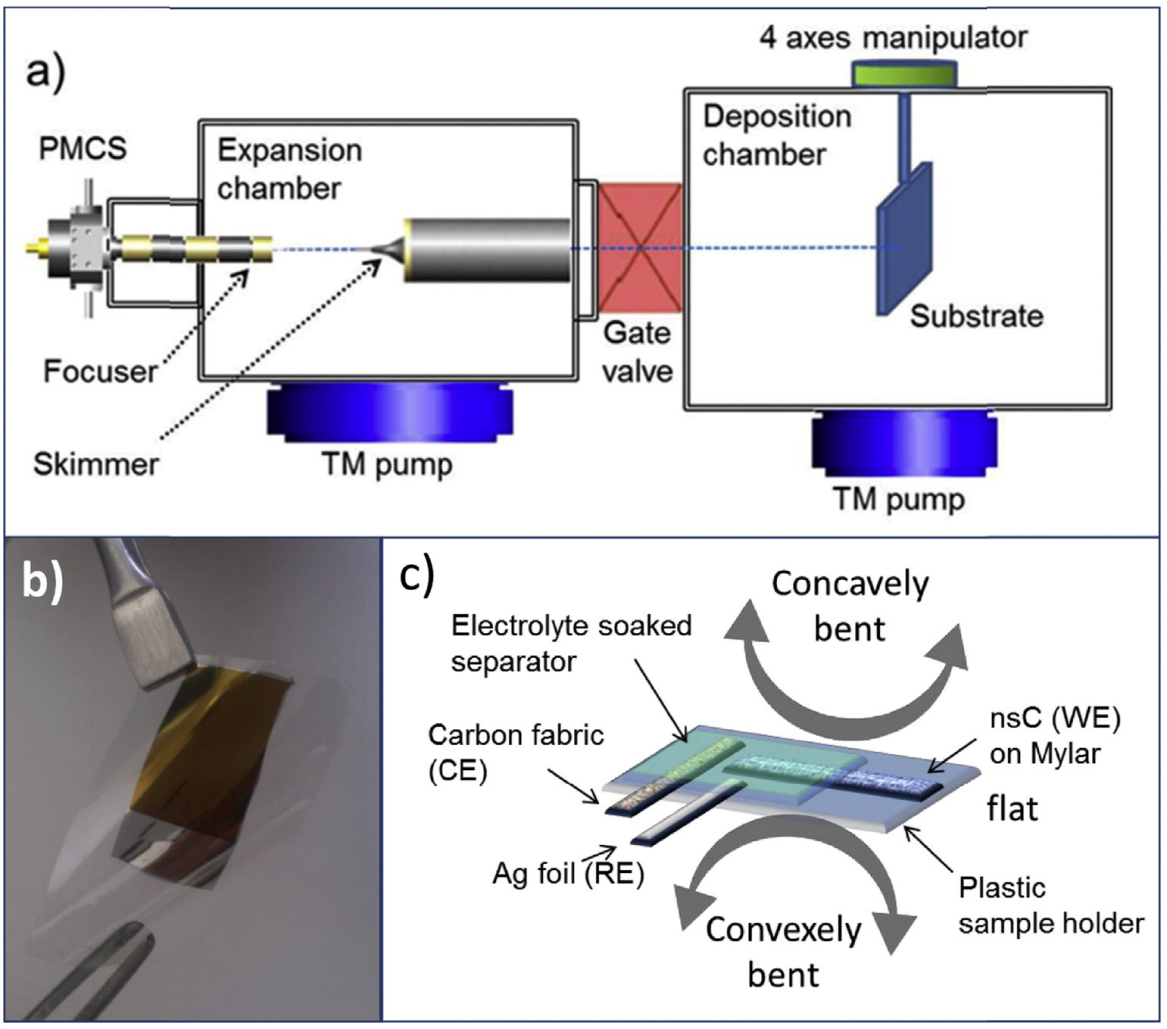
(a) Schematic representation of a SCBD apparatus equipped with a pulsed microplasma cluster source (PMCS), (b) image of 200 nm-thick nsC film (1 cm × 1.5 cm) deposited on Pt-coated Mylar substrate; (c) sketch of the experimental planar setup utilized for the electrochemical characterization of nsC electrodes.

The two cases discussed above of SC integration into different electrochemical systems like EGTs and MFCs highlight the key role played by high surface area carbons in the development of multifunction devices. Decoration of system components with nanostructured carbons will enable the design of the new, compact cell configurations with a positive impact on the volumetric energy performance and on device miniaturization. Miniaturization and integration of supercapacitive electrodes require the ability to fabricate porous thin films on a wide variety of substrates by high throughput techniques allowing a high control over the material properties (e.g. thickness and structure) and the compatibility with standard microfabrication processes. The Supersonic Cluster Beam Deposition (SCBD) fulfills these requirements [46], [47], [48]. This technique consists in the deposition at room temperature and under high vacuum conditions of nanoparticles accelerated in a free jet expansion to form a supersonic beam (Fig. 2a) [49]. Characterized by low kinetic energy of the deposited particles, SCBD produces a random stacking of clusters leading to nanostructured materials with high surface-to-volume ratio [50], [51]. The use of SCBD for the deposition of carbon clusters originates nanostructured carbon (nsC) thin films characterized by a disordered sp^2^ structure, a low density (ca. 0.5 g cm^−3^ [51]) and high specific surface area (ca. 700 m^2^ g^−1^ [52]) [53]. SCBD process enables the integration of nsC into capacitive electrodes avoiding binders and post-deposition treatments [51], [54] and was successfully employed for the fabrication of nsC based μSCs on glass and flexible polymeric substrates [47], [55]. μSCs fabricated on Mylar and employing *N*-trimethyl-*N*-propyl-ammonium bis(trifluoromethanesulfonyl) imide (N_1113_TFSI) IL as electrolyte feature cell voltage of 3 V with a capacitance density approaching 10 F cm^−3^ and maximum specific energy and power densities of 10 mW h cm^−3^ and 8–10 W cm^−3^ with long cycling stability over 2 × 10^4^ cycles [47]. The energy and power performance normalized to electrode area of 500 nm-thick μSCs are reported in Fig. 1. These energy storage properties address the requirements both for the switch-on of low-voltage EGTs making use of carbon gates and TransCap (Table S1).

Here, we discuss about the implementation and integration of nsC electrodes obtained by SCBD into miniaturized functional devices (e.g. biosensors) and energy harvesters, such as EGTs and MFCs, with the purpose of showing novel and sustainable approaches for the design of miniaturized autonomous systems. We report the results of the electrochemical characterization of flexible sub-μm thick nsC electrodes in different electrolytes, including ionic liquid (IL) and aqueous electrolytes. Specifically, N_1113_TFSI IL was selected because its wide electrochemical stability, good conductivity and low vapor pressure enable the development of high voltage μSCs operating above RT. The nsC behavior was investigated in aqueous NaCl and phosphate buffer (PBS) solutions because these electrolytes are typically used in bio-electrochemical devices, like bio-sensors and MFCs. The effect of reducing nsC thickness from 500 nm to 200 nm and of electrode bending, which is of interest for the design of flexible miniaturized devices, is reported. The interaction between bacteria and nsC is evaluated in view of the development of miniaturized, supercapacitive MFCs.

## 2 Experimental

### 2.1 nsC fabrication by SCBD

Cluster-assembled carbon films were deposited employing a SCBD apparatus equipped with a pulsed microplasma cluster source (PMCS) (a sketch of the SCBD apparatus is reported in Fig. 2a) [46], [50], [56]. A graphite rod, inserted in the PMCS, is sputtered by plasma which is confined by exploiting the pressure gradient produced by a jet of inert gas (He) impinging on its surface. Sputtered C atoms thermalize within the He and condense to form clusters. The mixture of clusters and inert gas exits the PMCS by expanding through a nozzle, thus forming a seeded supersonic beam of aerodynamically accelerated nanoparticles that are eventually collected on the substrate placed on the beam trajectory. As the cluster kinetic energy is low enough to avoid fragmentation upon deposition, a nanostructured carbon film retaining memory of the structural properties of the gas-phase aggregates is produced [50], [51]. The amount of deposited carbon is monitored in situ by a quartz microbalance placed closed to the substrates. NsC electrodes for electrochemical tests were fabricated at room temperature (RT) by the SCBD of carbon thin films on Mylar substrates previously coated by Ar ion sputtering with a thin platinum film serving as a current collector (Fig. 2b). NsC was also deposited on bare Mylar substrates to evaluate the adhesion of bacteria on cluster-assembled films.

### 2.2 Electrochemical tests

The electrochemical characterizations were performed using BioLogic VSP and VMP or PARSTAT 2273 (Princeton Applied Research) multichannel potentiostat/galvanostats in a planar 3-electrode setup (Fig. 2c). The nsC electrodes (working electrodes, WE) were coated by a fiber glass separator (Whatman,GF/F, dried before use) soaked with the electrolytes. A silver foil reference electrode (RE) and an activated carbon fabric counter electrode (CE; Spectracorp 2225, 2500 m^2^/g) were placed adjacent to the nsC electrode and ionically connected by the separator. A kapton tape was used to immobilize electrodes and separator on the sample substrate. The area exposed to the electrolyte of the 200 nm-thick nsC electrodes was 1 × 0.3 cm^2^.

The electrolytes were the ionic liquid N_1113_TFSI (99.5%, Solvionic, used as received) and aqueous solutions of 0.01 M NaCl and potassium 0.05 M PBS. The nsC response in IL was investigated in dry box (MBraun, Atmosphere, H_2_O and O_2_ < 0.1 ppm).

Electrode capacitance was evaluated by cyclic voltammetry (CV) and electrochemical impedance spectroscopy (EIS). The voltammetric capacitance is an average value over the swept electrode potential range. It has been obtained considering the slope of the integral of the voltammetric current over time vs. electrode potential. EIS measurements were performed in the 20 kHz–0.1 Hz frequency (f) range with 10 mV AC perturbation. The EIS capacitance (C_EIS_) was calculated by the imaginary component of the impedance (Zi) by the following eq.

(3) $${\text{C}}_{\text{EIS}}=1/\left(\left|\text{Zi}\right|2\text{πf}\right)$$

### 2.3 Bacteria interaction with nsC

In order to understand the interaction between bacteria and nsC, nsC deposited on Mylar substrate was fully immerged for 2 days in a sealed container containing a mixture of 50% in volume of activated sludge (from the Albuquerque Southeast Water Reclamation Facility, New Mexico, USA) and 50% in volume of 100 mM potassium phosphate buffer solution (PBS). Olympus epifluorescent microscope with DP71-IR microscope digital camera system and Microsuite image processing and analysis software was utilized to image bacteria on the nsC.

Particularly, LIVE/DEAD^®^ BacLight ™ Bacterial Viability kit was used to visualize microorganisms in the biofilms on the nsC. This particular kit allows the distinction of live bacteria that have plasma membranes still intact from dead bacteria having damaged external membranes. For live cell SYTO9 stain was used, while propidium iodide was used to identify dead cells. After 2 days, the nsC immerged in the mixture was removed, immerged few times into 50 mM PBS to wash out residual biomass and bacteria not interacting with the carbon surface and inserted into an Eppendorf tube containing 3 mL of PBS solution. 3 μL of SYTO9 stain and 3 μL of propidium iodide stain were inserted in the solution and incubated for 15 min. The nsC was then rinsed in PBS solution few times in order to wash out the excess of stain. The nsC on Mylar substrate was then placed on a microscope glass coverslip and images were taken. Particularly, 100× and 400× magnifications were used for clearly identifying and imaging bacteria on the nsC. To identify the fluorescence, U-MWIBA3 was used as filter. No dead cells were imaged. Images were also taken without the utilization of any particular filter.

## 3 Results and discussion

We have explored the capacitive response of sub-μm thick nsC electrodes deposited on Mylar substrate in different electrolyte media and in flat and bent conditions. Specifically, we performed CV and EIS measurements in the N_1113_TSI IL, of interest for high voltage and temperature applications, and in 0.01 M NaCl and 0.05 M potassium phosphate buffer (PBS) aqueous solutions that are of interest for bio-electrochemical systems.

**Fig. 3:**
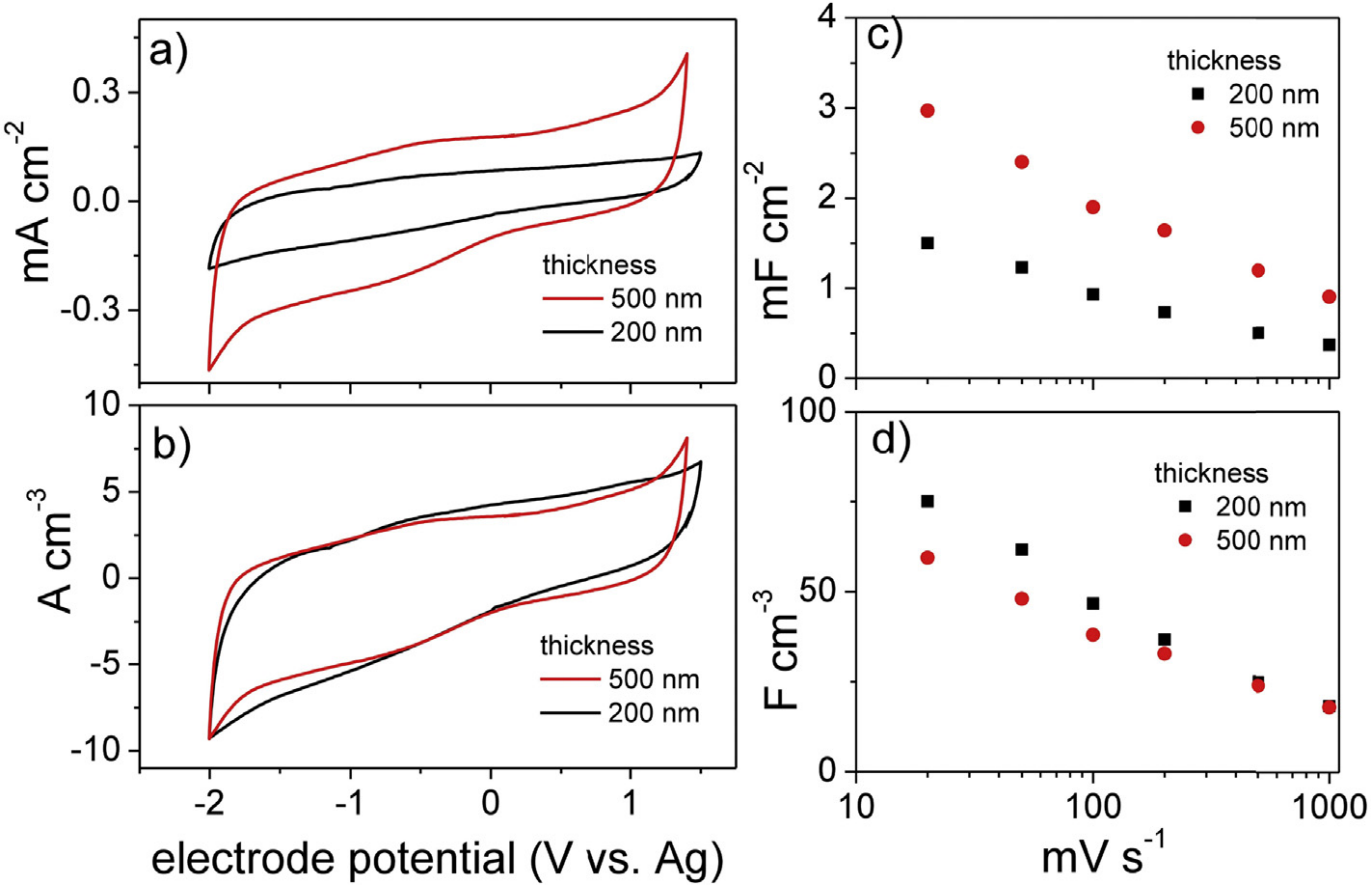
CV study of 200 nm (200-nsC) and 500 nm (500-nsC)-thick electrodes in the IL N_1113_TFSI. Voltammograms at 100 mV s^−1^ with currents normalized to electrode area (a) and volume (b); area (c) and volumetric (d) electrode capacitances at different scan rates.

Fig. 3a-b compare the voltammetric behavior at 100 mV s^−1^ of 200 nm (200-nsC) and 500 nm (500-nsC)-thick electrodes in the ionic liquid N_1113_TFSI. The voltammetric currents are normalized both to the electrode area and volume.

**Fig. 4:**
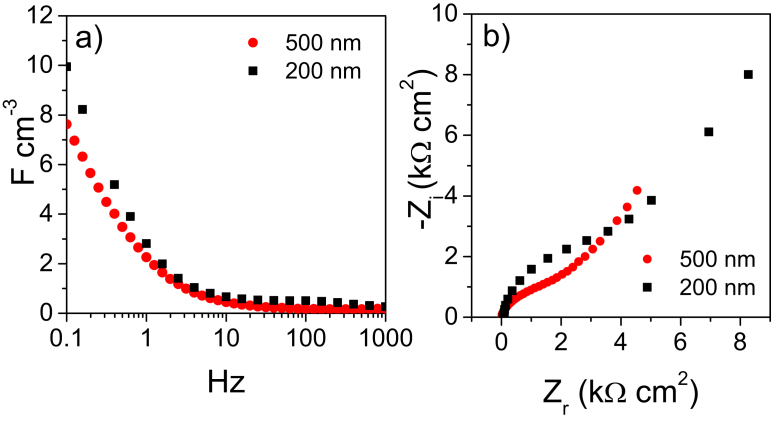
EIS study of 200 nm (200-nsC) and 500 nm (500-nsC)-thick electrodes in the ionic liquid N_1113_TFSI. (a) Volumetric electrode capacitance vs frequency plot; (b) Nyquist diagrams with impedances normalized to electrode area.

The electrodes featured a capacitive response within −2 V and 1.5 V vs Ag which supports the viability of 3 V- μSC. The not-perfect box shape of the CVs is related to the asymmetric capacitive response in the positive and negative potential domains of the nsC electrodes [47]. Fig. 3a shows that the CV current density linearly scales with electrode thickness. Indeed, the currents normalized to electrode volume reported in Fig. 3b overlap almost perfectly. The analysis of the CVs performed at scan rates varying from 20 mV s^−1^ up to 1 V s^−1^ (Fig. S3) provided the areal capacitances that decreased from 1.5 mF cm^−2^ to 0.4 F cm^−2^ for 200-nsC and from 3 mF cm^−2^ to 0.9 mF cm^−2^ for 500-nsC (Fig. 3c-d), respectively. The volumetric capacitances at the lowest scan rate were 75 F cm^−3^ for 200-nsC and 60 F cm^−3^ for 500-nsC and decreased to 20 F cm^−3^ at 1 V s^−1^ for both the electrodes. The capacitance trend with scan rate reflects the electrode rate behavior and can be explained by the Bode and Nyquist plots reported in Fig. 4. The Bode plots are reported in terms of volumetric capacitance vs frequency in the 0.1 Hz–1 kHz range (Fig. 4a). For both 200-nsC and 500-nsC, the capacitive response is exhibited below 1 Hz and limit capacitance is not reached at 0.1 Hz. The Nyquist plots consist of a high frequency semicircle and a low frequency 45° tail at low frequencies. The Z_r_-axis intercept of the plots at the highest frequency is the uncompensated electrode resistance which depends on cell setup and includes electronic (current collector) and ionic terms (electrolyte bulk resistance). The high frequency semicircle originates from the resistive and capacitive processes involved in the double layer-charging process, namely contact resistance (grain boundary) and contact capacitance between carbon particles and between nsC and current collector. For both the electrodes investigated, the main contribution to the areal impedance is represented by the Warburg low frequency tale which originates from a slow ion diffusions into the porous nsC layer and explains the capacitance trends vs. CV scan rate (Fig. 3 c, d) and EIS frequency (Fig. 4a).

The above reported results demonstrate that SCBD can be exploited for the development of very thin, IL-based μSC that can operate at 3 V. The EIS study indicates that the nsC/electrolyte interface has to be ameliorated and ion diffusion has to be improved in order to lower the Warburg impedance. This will positively impact on the equivalent series resistance (ESR) and power of the μSC and might be pursued by the use of low-viscosity electrolyte formulations. The areal maximum energy stored by μSCs featuring 200-nm electrodes can be calculated by the equation

(4) $${\text{E}}_{\text{max}}=½{\;\text{C}}_{\text{μSC}}{\text{V}}^{2}$$

C_μSC_ of a symmetric device can be reasonably set equal to half the electrode capacitance (in mF). The areal and volumetric C_μSC_ should consider the footprint area and volume of the two electrodes and would result 0.35 mFcm^−2^ (i.e. 1/4 the areal electrode capacitance obtained from the voltammetric value at 10 mV s^−1^) and 18 Fcm^−3^ (i.e. the areal C_μSC_ divided per the electrode thickness). These data bring about an E_max_ of 0.45 μW h cm^−2^ and 23 mW h cm^−3^, values that are higher than those that we previously reported with thicker electrodes [47]. Furthermore, the projected E_max_ well competes with the performance of carbon-based μSCs reported in literature with the advantage of being obtained with much thinner electrodes. Indeed, SCBD permits to control and reduce electrode thickness below the micrometric scale, a scale typically reported for carbon-based μSCs [57], [58].

Notably, the projected energy performance of the 200 nm-μSCs satisfies the energy requirements of EGTs (cfr. Table S1) and supports the idea that SCBD can be adopted to develop planar device architectures in which a μ-energy storage unit is embedded into a μ-electronic components. Furthermore, the capacitive response of nsC electrodes make them exploitable as gates of low-voltage EGTs and TransCaps. Indeed, the channel doping charges Q_ch_ reported in Table S1 are in the 100–300 μC range and correspond to the charge that has to be stored/delivered by the EGT carbonaceous gate. This charge can be accomplished by 200-nsCs (that feature ca. in 1 mF cm^−2^) within a narrow electrode potential range (ΔV_gate_) of 0.1–0.3 V, thus enabling a narrow ΔV_gs_ (see Supplemental Information).

**Fig. 5:**
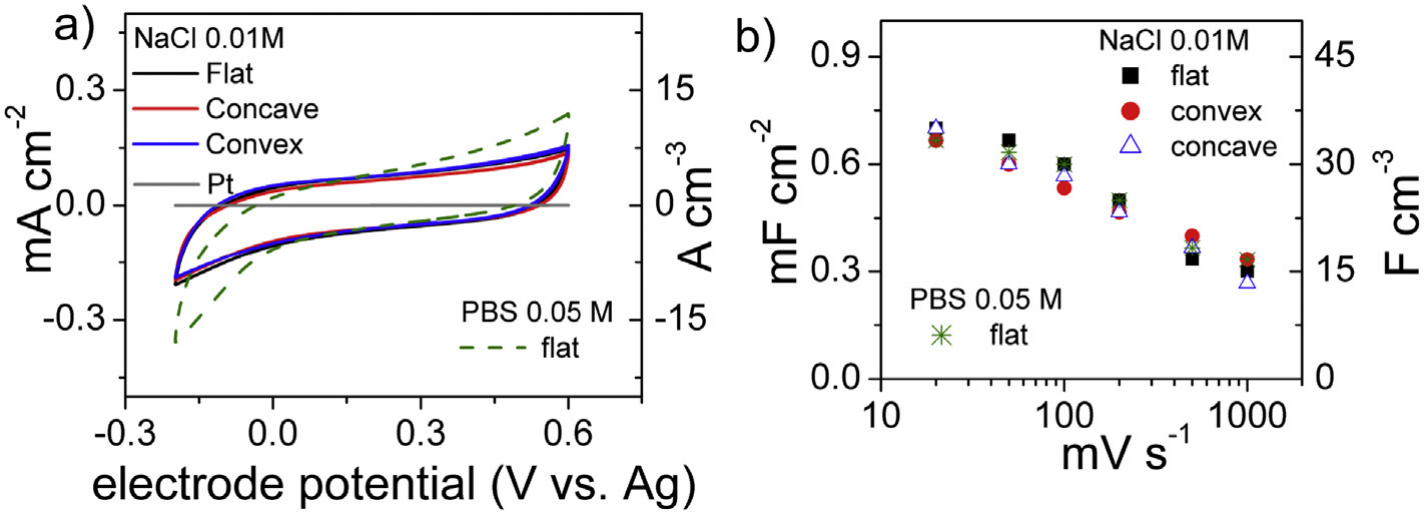
CV study of 200 nm (200-nsC) electrodes in 0.01 M NaCl (flat and concavely or convexly bent) and 0.05 M PBS aqueous solutions. (a) Voltammograms at 100 mV s^−1^ with currents normalized to electrode area volume; (b) areal and volumetric electrode capacitances at different scan rates.

The sub-1V working regime of EGTs and TransCaps assembled with carbon gates enables aqueous electrolyte gating, provided that channel materials are water compatible. This is of great interest for bioelectronic implants applications. It has been demonstrated that current modulation of organic electrochemical transistors (OECTs) based on PEDOT:PSS (a biocompatible material) are enhanced if PEDOT:PSS gate is substituted with a commercial activated carbon gate. This was proven using an aqueous solution of 0.01 M NaCl as electrolyte [26]. Concerning this view and supporting the idea of exploiting SCBD for developing flexible OECTs we carried out a voltammetric characterization of 200-nsC electrodes in 0.01 M NaCl aqueous solution and the results are reported in Fig. 5. Fig. 5a shows the CVs at 100 mV s^−1^ of flat and of concavely and convexly bent 200-nsC electrodes. The three CVs overlap and demonstrate the good mechanical properties of the films. Fig. 5b shows that the areal capacitance decreases from 0.7 mF cm^−2^ to 0.3 mF cm^−2^ for scan rates ranging from 10 mV s^−1^ to 1 V s^−1^ (Fig. S4); the corresponding volumetric capacitances are 35 F cm^−3^ and 15 F cm^−3^.

**Fig. 6:**
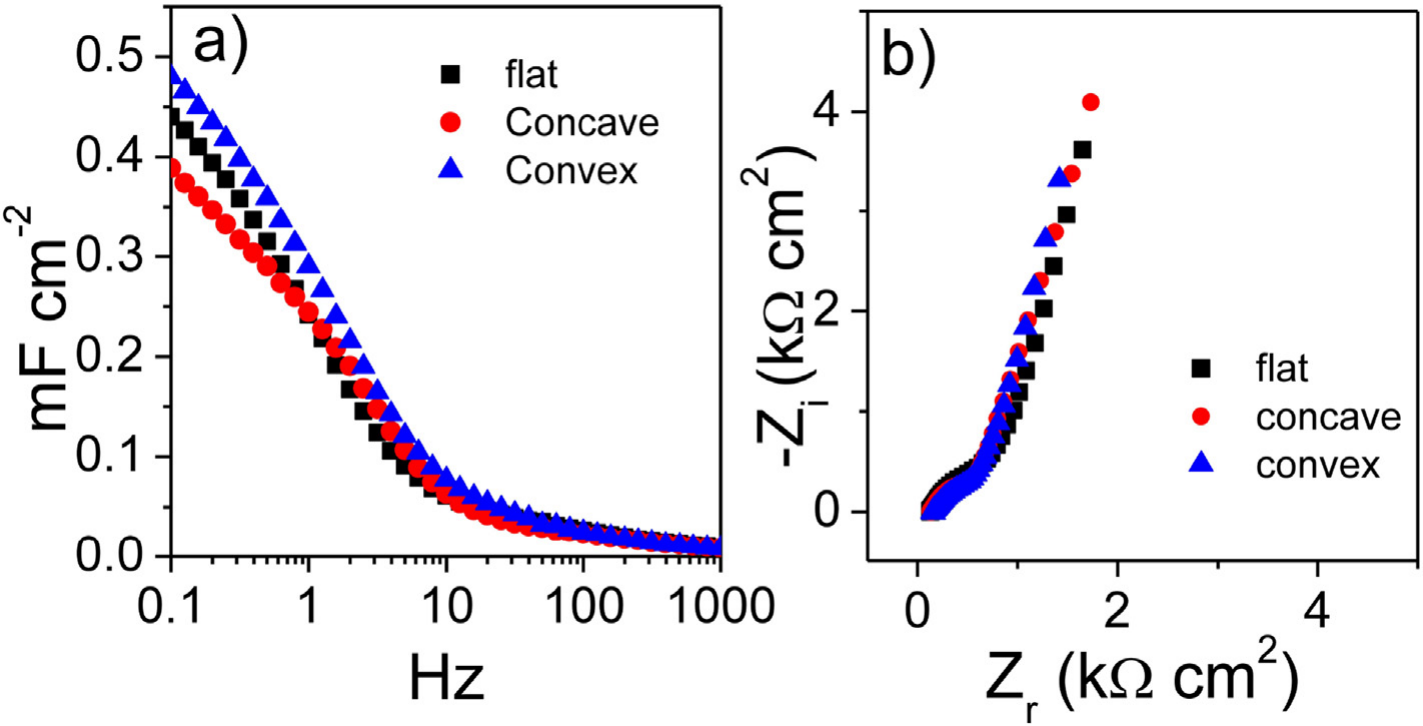
EIS study of 200 nm (200-nsC) electrodes flat and concavely or convexly bent in 0.01 M NaCl. (a) Volumetric electrode capacitance vs frequency plot; (b) Nyquist diagrams with impedances normalized to electrode area.

The capacitive response of 200-nsC is lower in 0.01 M NaCl aqueous electrolyte compared to N_1113_TFSI IL. This might be due to the lower conductivity (2.0 mS cm^−1^ for 0.01 M NaCl and 3.3 mS cm^−1^ forN_1113_TFSI, at RT [59]) and the lower ionic concentration of the aqueous electrolyte with respect to the ionic liquid (3.8 M, based on a formula weight of 382 g mol^−1^ and a density of 1.44 g mL^−1^). Moreover, the different size of hydrated ions and of solvent-free ionic liquid ions may play a role in the capacitive response. Despite the lowest capacitance, the 200-nsC rate behavior is superior in aqueous electrolyte than in IL. Indeed the Bode plot reported in Fig. 6a shows that in 0.01 M NaCl the nsC electrodes feature a capacitive behavior below 10 Hz and capacitance approaches the limiting value at 0.1 Hz. The high frequency semicircle diameter is smaller than what observed in IL and the low frequency impedance is approaching the pure capacitive response (the phase angle is 70°). These features suggest that the double-layer charging process is faster and less affected by ion diffusion in the aqueous electrolyte than in IL, which might be explained with the lower viscosity of the former solution (ca. 1 cP) with respect to N_1113_TFSI (79 cP, at RT [59]).

Despite the good EIS response, the electrode potential window that can be exploited for electrode charge/discharge is lower than 1 V (see Fig. 5a). This renders μSC with 200 nsC electrodes and 0.01 M NaCl solution (that would exhibit a maximum cell voltage of 1 V) not competitive with those featuring N_1113_TFSI (that are capable to achieve 3 V, Fig. 3a). On the other hand, the above reported good rate and capacitive responses of the nsC electrode in 0.01 M NaCl can be exploited to assemble flexible, aqueous EGTs featuring a carbonaceous gate.

The electrochemical response of 200 nsC electrodes was also investigated in a phosphate buffer solution (0.05 M PBS, solution conductivity 7.3 mS cm^−1^ at 24 °C) and the results are reported in Fig. 5 and Figure S4b. PBS is commonly used to mimic physiological conditions in biosensing studies and to stabilize pH and increase the solution conductivity in MFCs. The voltammetric and capacitive response of 200-nsC electrodes are very similar to what observed in 0.01 M NaCl and of interest for bioelectrochemical miniaturized systems.

While the areal capacitance of 200-nsC is one order of magnitude lower than the value exhibited by the glass bottle supercapacitive MFC-AdE (of ca. 10 mF cm^−2^), the good volumetric capacitance opens the way to the use of SCBD to design miniaturized and supercapacitive MFCs.

**Fig. 7:**
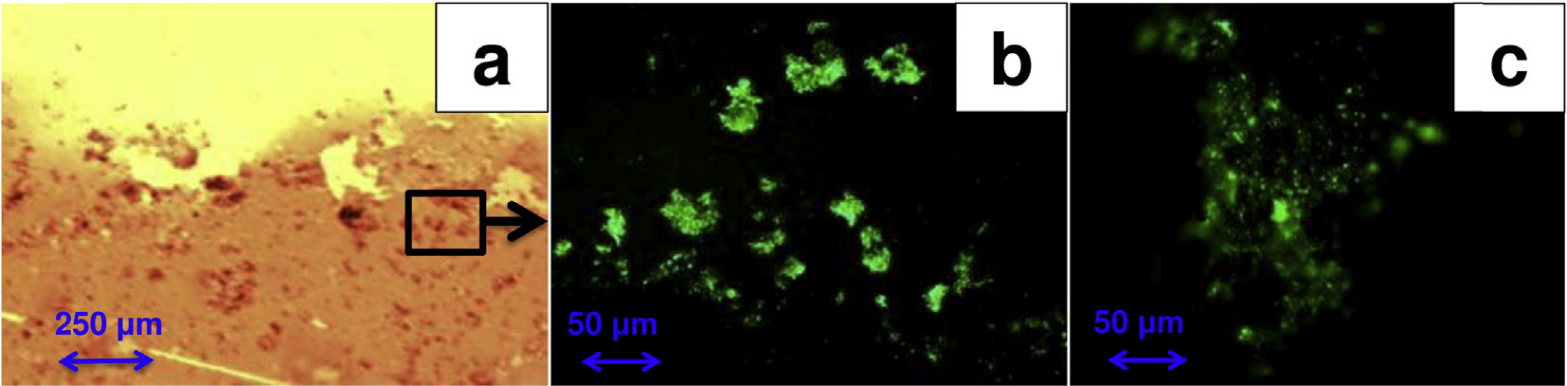
Microscope images of: bacteria attached on nsC and Mylar at 100× magnification with no filter utilized (a); stained bacteria attached on nsC using fluorescence U-MWIBA3 filter (b and c).

Bacteria interaction and attachment with the anode electrode surface is a fundamental feature for biofilm formation and development and consequently successful MFC operation. We, therefore, tested the affinity and interaction between bacteria and nsC material immerging the nsC carbon layer (280 nm) deposited on Mylar substrate into a solution containing 50% of activated sludge and 50% of 0.1 M PBS. Images showed interesting results and particularly, Fig. 7a shows distinctively the nsC (orange), the Mylar substrate (yellow) and the bacteria (darker dots or aggregates). Interestingly, it seems that bacteria prefer to attach to the carbon substrate rather than the Mylar substrate. In fact, only few bacteria are detected on the Mylar probably washed out during the staining procedure. Bacteria from the activated sludge showed to have higher interaction and more biocompatibility and bioaffinity to the modified carbon substrate. Those results indicate the possibility of utilizing the nsC as anodes for μMFC or μSC-MFC. Further images have been taken to enhance the fluorescence of the stained bacteria using particular filter (U-MWIBA3) over the nsC surface (Fig. 7b,c). In this case, the magnification was increased to 400× in order to show the bacteria agglomerates and not distinctive bacteria formed on the surface.

## 4 Outlook and conclusions

Flexible supercapacitive electrodes can be fabricated at room temperature by the Supersonic Cluster Beam Deposition (SCBD) of nanostructured carbon (ns-C) films on polymeric substrates like Mylar. This process is particularly interesting for the development of planar and flexible devices based on highly porous carbon thin films. Compared to other carbon materials recently employed as electrode in miniaturized supercapacitors (e.g. carbon nanotubes, carbide derived carbons, onion like carbons and graphenes), nsC has the key advantage of being easily deposited at room temperature onto almost any kind of substrate with high control over carbon thickness (from few tens of nanometers up to micrometers), and avoiding binders and complicated and expensive fabrication steps.

The good volumetric capacitance of nsC electrodes can be exploited for different devices, that, if properly designed and connected could bring about a miniaturized autonomous system.

μSCs fabricated by SCBD and working with ILs exhibit performances that address the typical requests of a number of devices requiring microscale energy storage. The use of ILs enables μSC operation at 3 V and above room temperature which is particularly important in view of the integration of μSCs with electronic components operating at high current regimes, where electrical power is dissipated into heat. Improvements of the energy storage performances can be reasonably expected as a consequence of the optimization of the device design, of the ns-C thickness and of the choice of the electrolytes.

SCBD can be used to process sub-1V EGT components and to design novel architectures for new multifunction-energy storage elements. Specifically, nsC electrodes can be used as gates in EGTs and TransCap assembled with both organic and aqueous electrolytes, the latter of interest for biosensing applications. Work is in progress on the development of flexible EGTs e TransCaps prepared by SCBD.

The capacitive response of nsC electrodes in neutral environment and the compatibility of the nsC layer with bacteria also pave the way towards the use of SCBD for the design of miniaturized anode for MFCs or supercapacitive MFCs. This is an innovative way of thinking energy harvesting and simultaneous wastewater treatment in systems in which boosting up energy output is required for practical and real applications.

Our study demonstrates that the development of miniaturized autonomous systems that exploit the supercapacitive features of materials and devices and that are processed by sustainable processes, like SCBD, is feasible. Our ambition is to contribute to new research directions on the exploitation of capacitive material into self- and low-power systems.
