# Supplementary material for: Miniaturized supercapacitors: key materials and structures towards autonomous and sustainable devices and systems
Source: J Power Sources. 2016 Sep 15;326:717–25. doi: 10.1016/j.jpowsour.2016.04.131 (PMC4997707; doi:10.1016/j.jpowsour.2016.04.131)
Supplement: Supplementary file 1 [file mmc1.docx]

**Miniaturized Supercapacitors: key materials and structures towards autonomous and sustainable devices and systems**

Francesca Soavi^1,*^, Luca Giacomo Bettini^2^, Paolo Piseri^2^, Paolo Milani^2^, Carlo Santoro^3^, Plamen Atanassov^3^, Catia Arbizzani^1^

^1^ Department of Chemistry “Giacomo Ciamician”, Alma Mater Studiorum - Università di Bologna, Via Selmi, 2, 40126 Bologna, Italy.

^2^ CIMaINa and Physics Department, Università degli Studi di Milano,Via Celoria 16, 20133 Milano, Italy

^3^ Department of Chemical & Biological Engineering, Center for Micro-Engineered Materials (CMEM), University of New Mexico, Albuquerque, NM 87131, USA.

1. **Supplemental experimental and methods**

*1.1 EGT structure and test*

Drain and source contact electrodes of Ti/Au, 5/40 nm/nm, were deposited on cleaned SiO_2_ substrates by e-beam physical vapor deposition and patterned using photolithography to form single channel electrodes.

Organic channel films were prepared by spin coating of solutions based on poly[2-methoxy-5-(20-ethylhexyloxy)-1,4-phenylene vinylene] (MEH-PPV, Sigma Aldrich, 55 kDa) as in refs. [1]. Inorganic WO_3_ channels were directly prepared by sol−gel on prepatterned substrates [2].The gate electrodes were prepared by drop casting an ink of activated carbon (PICACTIF SUPERCAP BP10, Pica) and polyvinylidene fluoride (PVDF, KYNAR HSV900) binder in N-methyl pyrrolidone (NMP, Fluka, >99.0%) on carbon paper (Spectracorp 2050).

Channel and gate were separated by a Durapore GVHP filter membrane (9 mm x 4 mm x 125 µm) soaked with the electrolyte. Specifically, ionic liquids (ILs) based on the [TFSI] anion and 1-ethyl-3-methylimidazolium (EMIM), 1-butyl- 3-methylimidazolium (BMIM), 1-butyl-1-methylpyrrolidinium (PYR_14_), or N_1113_ cations (dried before use) were used for EGTs based on MEHPPV or WO_3_ channels that were tested in a N_2_ glovebox (H_2_O, O_2_ < 5 ppm). The electric characteristics of these devices were measured using a B1500A Agilent semiconductor parameter analyzer.

*1.2 MFC materials and configuration*

The anode of the MFC was a high surface area carbon brush composed of titanium wire core twisted on the carbon fibers [3]. The carbon brush had a diameter of 3 cm and the height was 3 cm disposed in cylindrical way. The anode was colonized by mixed bacterial cultures from civil wastewater that was enriched over several months leading to the establishment of electroactive biofilm. The MFC cathode was in air-breathing configuration (one side facing the solution and one side facing the atmosphere). The cathode was made by mixing and pressing (2 mT for 5 minutes) high surface area activated carbon (AC, ≈900 m^2^g^-1^) with polytetrafluoethylene (PTFE) and carbon black (CB) [4]. The additional electrode (AdE) was a carbon brush with diameter of 2 cm and height of 2 cm. The AdE was short-circuited with the cathode electrode and immersed in the same electrolyte solution. The working solution was a 50:50 mixture between activated sludge and 0.1M phosphate buffer saline (PBS) that guarantees high ion concentration and circumneutral pH [5].

*1.3 Electrochemical tests*

The electrochemical characterizations of the µSC, MFCs and EGTs were performed using BioLogic VSP and VMP or PARSTAT 2273 (Princeton Applied Research) multichannel potentiostat/galvanostats.

The equivalent series resistance (ESR), capacitance, practical energy (E) and power (P) delivered during discharge of the different devices were evaluated by galvanostatic test (except were differently indicated) as follows.

The equivalent series resistance of the cell (ESR) was evaluated by the ohmic drop ΔV_ohmic_ at the beginning of discharge by the eq. ESR = ΔV_ohmic_/i, where i is the discharge current density. The cell capacitance was calculated by dividing the current per the slope of the cell voltage vs. the time.

E (in Wh cm^-2^) and P_av_ were calculated by the integral of the cell voltage over time by using the eqs. (1) and (2):

E = i ∫ V dt/3600 (1)

P = E _av_ / Δt, (2)

where Δt is the discharge time.

**2. Supercapacitor integrated systems: the concepts**

*2.1 Integration of Supercapacitors into electronic components: EG transistors and TransCap*

EGTs consist of source (S) and drain (D) electrodes and a semiconductor channel in ionic contact with a gate electrode via an electrolyte solution. The gate-source voltage (V_gs_) modulates the current flowing in the channel between the drain and source electrodes (I_ds_). Current flows through the channel by application of a voltage (V_ds_) between drain and source (Figure S1a).

The channel/electrolyte/gate stacking of EGTs can be viewed as a 2-electrode electrochemical cell where |V_gs_| is the difference of the gate (V_gate_) and channel (V_ch_) electrode potentials (vs. a given reference)

|V_gs_| = |V_gate_-V_ch_| (1)

The electrode potential of the gate vs. channel material (and viceversa) has to be properly set to optimize current modulation in EGTs based on redox channels.

When a V_gs_ bias is applied, both channel and gate electrode potentials change as described by Figure S1.

Eqs. (1) suggests that even the electrochemical behaviour of the gate is affecting EGTs current modulation. Developing a low voltage (low V_gs_) EGT, which positively affect power consumption and device stability, requires that the redox activity of the channel has to take place at low potentials vs. the gate potential, i.e. V_ch_ ≅ V_gate_, and that the channel and gate potential excursions are as narrow as possible. Being a bulk process, the doping of redox channels involves a high doping charge (Q_ch._) and pseudocapacitance (C_ch_), which can be up to two orders of magnitude larger than those involved in the electrostatic polarization of a flat surface/electrolyte interface. Channels with high pseudocapacitance (C_ch_ ≅ 100-200 F g^-1^) typically exhibit narrow ΔV_ch_, indeed

C_ch_= Q_ch_/ΔV_ch,_  (2)

The channel doping charge has to be counterbalanced by the gate, i.e.

Q_ch_=Q_gate,_  (3)

and

C_ch_ΔV_ch_ = C_gate_ΔV_gate,_  (4)

Conventional metal gates like Pt, of low gate capacitance (C_gate_), are not capable to effectively counterbalance the doping processes occurring at redox channels within a narrow ΔV_gate_. On the other hand, gate electrodes based on high surface area carbons with high double-layer capacitance (≅ 100 F g^-1^), can store/deliver a noticeable amount of charge Q_gate_ by a fast and reversible electrostatic process within a relatively low ΔV_gate._ , thus enabling an overall narrow ΔV_gs_ [6]. These concepts are described in Figure S1a. The electric response of the organic and inorganic EGTs making use of high surface area carbon gate and ionic liquid (ILs) electrolytes are summarized in the first columns of Table S1 in terms of output characteristics (I_ds_ at the saturation threshold V_ds_ and ON/OFF current) and of the highest V_gs_ required to completely dope the channel with a Q_ch_ doping charge (from transfer experiments).

The energy required to drive EGTs (E_EGT_) includes the contributions to dope the channel (E_gs_) and to let the current flowing through the channel (E_ds_) over a certain time (t), i.e.

E_EGT_= E_gs_ + E_ds_  (5)

The doping energy is related to V_gs_ and Q_ch_ by

E_gs_= |V_gs_ Q_ch_| (6)

This is why low-V_gs_ EGT can be considered low-power devices.

E_ds_ depends on the power dissipated through the channel, in turn related to I_ds_ and t, i.e.

E_ds_= P_ds_ t (7)

where

P_ds_= |I_ds_ V_ds_| (8)

Table S1 reports the energy and power demand of EGTs with polymer and inorganic channels, electrolytes based on ionic liquids and propylene carbonate (PC) and high surface area carbon gate.

The capacitive EGT channel and carbon gate components reversibly store charge at given V_gs_, so that they permit to reversibly store electrochemical energy E_gs_ like in a hybrid μSC. The energy used to dope the channel (E_gs_) is stored and, then, deliverable upon the EGT switch OFF. The percentage of stored energy is E_stored_ = 100 E_gs_/ E_EGT_ and is reported in the last column of Table S1 for the different EGTs here considered. I_ds_ and I_gs_ and, hence, the EGT power demand depend on key size parameters like the channel thickness and area. Therefore, data in Table S1do not pretend to be considered as a benchmark but could be further ameliorated by design optimization. Table S1 even reports the electrical and energy performance of Transcap, that integrated an EGT and a μSCs at materials level [7]. TransCap featured an MEHPPV channel, a gate based on commercial activated carbon layer deposited on carbon paper and the ionic liquid N_1113_TFSI. Figure S1b shows the trends of the power demand for current flow through the channel of TransCap and of the power stored upon channel doping (P_gs_= I_gs_ V_gs_) and delivered upon channel undoping under three repeated switch ON and OFF steps of TransCap. The integration of P_gs_ over time provide and E_gs_ of ca 0.02 µWh stored during switch on and delivered during the following switch OFF with an efficiency of 99.5%.


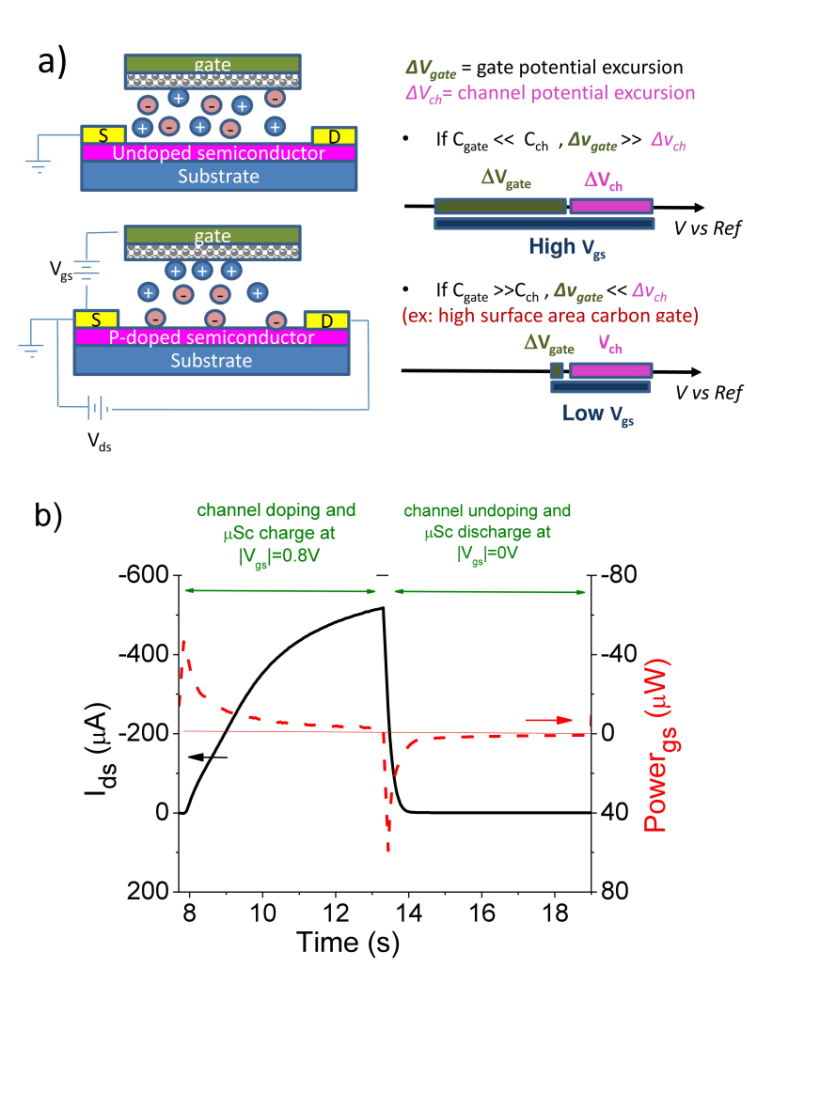


Figure S1. a) Scheme of low-voltage EGT operation. b) Repeated Switch ON and OFF steps of TransCap. Power demand for I_ds_ flow through the channel of TransCap (P_ds_, left axis), and power stored upon channel doping and delivered upon channel undoping (P_gs_ = I_gs_ V_gs_) right axis).

Table S1. Electrical and Energy demand of EGTs and of TransCap making use of high surface area carbon gate and ionic liquid-based electrolytes. The channel and electrolyte components of the devices are reported in the first two columns. Q_ch_ is the doping charge evaluated upon the voltammetric scan of V_gs_ up to the value reported in the Table during the transfer test at a V_gs_ sweeping rate of 10 mV s^-1^; the ON/OFF ratio is deduced from the forward step of the transfer curves. I_ds_ is the saturation current flowing through the channel at the V_ds_ reported in the Table and evaluated by the output tests at the highest |V_gs_|. The energy required to dope the channel (E_gs_) , the power (P_ds_) and energy (E_ds_, for 5 s switch-on time) needed to let the current flowing through the channel, and the total energy cost to drive the EGTs (E_EGT_) are calculated by eqs. 5-8. The last column reports the percentage of stored energy (E_stored_). For TransCap the Q_ch_ and energy and power figures have been obtained from Figure S1b.

| Channel | Electrolyte | \|Q_ch_\|  µC | \|V_gs_\|  V | ON/  OFF | I_ds_  µA | \|V_ds_\| | E_gs_  µWh | P_ds_  µW | E_ds_  µWh | E_EGT_  µWh | E_stored_  % |
| --- | --- | --- | --- | --- | --- | --- | --- | --- | --- | --- | --- |
| EGT |  |  |  |  |  |  |  |  |  |  |  |
| MEHPPV | EMIMTFSI | 170 | 1.0 | 2-3.10^3^ | 1200 | 0.25 | 0.05 | 300 | 0.40 | 0.45 | 11 |
|  | BMIMTFSI | 230 | 1.0 | 2-3.10^3^ | 2500 | 0.25 | 0.07 | 625 | 0.80 | 0.87 | 8 |
|  | PYR_14_TFSI | 180 | 1.0 | 2-3.10^3^ | 1200 | 0.25 | 0.05 | 300 | 0.40 | 0.45 | 11 |
|  | PC-PYR_14_TFSI | 95 | 1.0 | 1.8.10^3^ | 170 | 0.15 | 0.03 | 25 | 0.03 | 0.06 | 50 |
| WO_3_ | PYR_14_TFSI | 300 | 1.2 | 100 | 500 | 0.7 | 0.10 | 350 | 0.49 | 0.59 | 17 |
| TransCap |  |  |  |  |  |  |  |  |  |  |  |
| MEHPPV | N_1113_TFSI | 70 | 0.8 | 2.10^3^ | 500 | 0.3 | 0.02 | 150 | 0.2 | 0.22 | 9 |

Electric characteristics from refs. [1, 2, 7].

*2.2 Integration of Supercapacitors into MFCs*

Microbial fuel cells (MFCs) are innovative bioelectrochemical systems specifically devoted to convert organic compounds into electricity. The conversion from organics into electricity happens via the contribution of electroactive biofilm on the anode electrode that oxidize the organics as carbon sources releasing the electrons directly to the electrode itself. The red-ox reaction is closed at the cathode electrode in which an electron acceptor is reduced. The cell voltage in open circuit conditions (OCV) at pH of 7.5 is 1.12 V that is the difference between the oxygen reduction reaction (ORR) potential (≈+0.59 V vs Ag/AgCl sat. KCl) and the acetate oxidation potential (≈-0.53 V vs Ag/AgCl sat. KCl). In a working MFC, the anode open circuit potential (OCP) is similar to the theoretical value while at the cathode OCP is usually 0.2-0.3 V lower than the theoretical value due to ohmic losses and to the low activity of the catalysts at circumneutral pH [8].

In a working MFC, electrogenic microorganisms colonize the anode electrode and oxidize the available substrate (i.e., the electron donor). In a MFC, the anaerobic oxidation releases electrons to the anode directly or through mediators [9]). The air-breathing configuration of the cathode allows the establishment of a three phases interface (TPI) and consents the presence of oxygen in the cathode preserving aerobic conditions. In rest, the bio-anode and oxygen cathode redox couples move the electrodes at potentials that are at more negative and positive, respectively, than the typical value exhibited by not polarized carbons in de-aerated aqueous electrolytes (near 0 mV vs Ag/AgCl [10]). This causes the creation of a potential difference and the consequent electrode polarization along with the formation of electrochemical double layers (EDLs) at the electrode interfaces without the application of any external voltage bias. Therefore, an internal, charged EDLC is formed and anode and cathode of the MFC can be directly used as the negative and positive electrodes [5] (Figure S2a)

Figure S2b shows the cell voltage and positive and negative electrode potential profiles (dashed lines) of an MFC with a brush anode and an activated carbon (AC) based cathode upon 2 mA cm^-2^ (i, normalized to the cathode area). The typical plateau related to the MFC redox processes is absent and a linear decrease of cell voltage is evident. This transient profile represents the double layer discharge of the polarized carbon electrodes of the internal SC. The cell capacitance is 12 mF cm^-2^ and the equivalent series resistance (ESR) of the cell evaluated by ΔV_ohmic_ was 210 ohm cm^2^.

In order to decrease the cathode ohmic resistance and surmount the existing cathode capacitive weaknesses, an additional electrode based on a small carbon brush coated with AC was short-circuited with the cathode and inserted in the same solution [5]. The short-circuit allowed to the AdE to have the same potential of the cathode. With this strategy, the anode of the MFC was used as negative electrode of the SC and the additional electrode (AdE) was used as positive SC electrode. Figure S2b reports the cell voltage and electrode potential profiles of this system (labelled with MFC-AdE, solid lines) under 2 mA cm^-2^ discharge. MFC-AdE featured the same OCV of the MFC. During fast discharge, the current flowed through the lowest resistance electrodes, i.e. the MFC anode and AdE and consequently the MFC cathode was excluded thus reducing the cell ohmic drop. ESR was contained within 50 Ω cm^2^, in turn enabling longer discharges at higher currents. Figure 3c shows the cell voltage trend during two discharges at 4 mA cm^-2^ each followed by a rest period. Notably, during rest, MFC-AdE recovered the cell voltage value exhibited before discharge within 20 s, thus demonstrating the fast self-rechargeability of the SC.


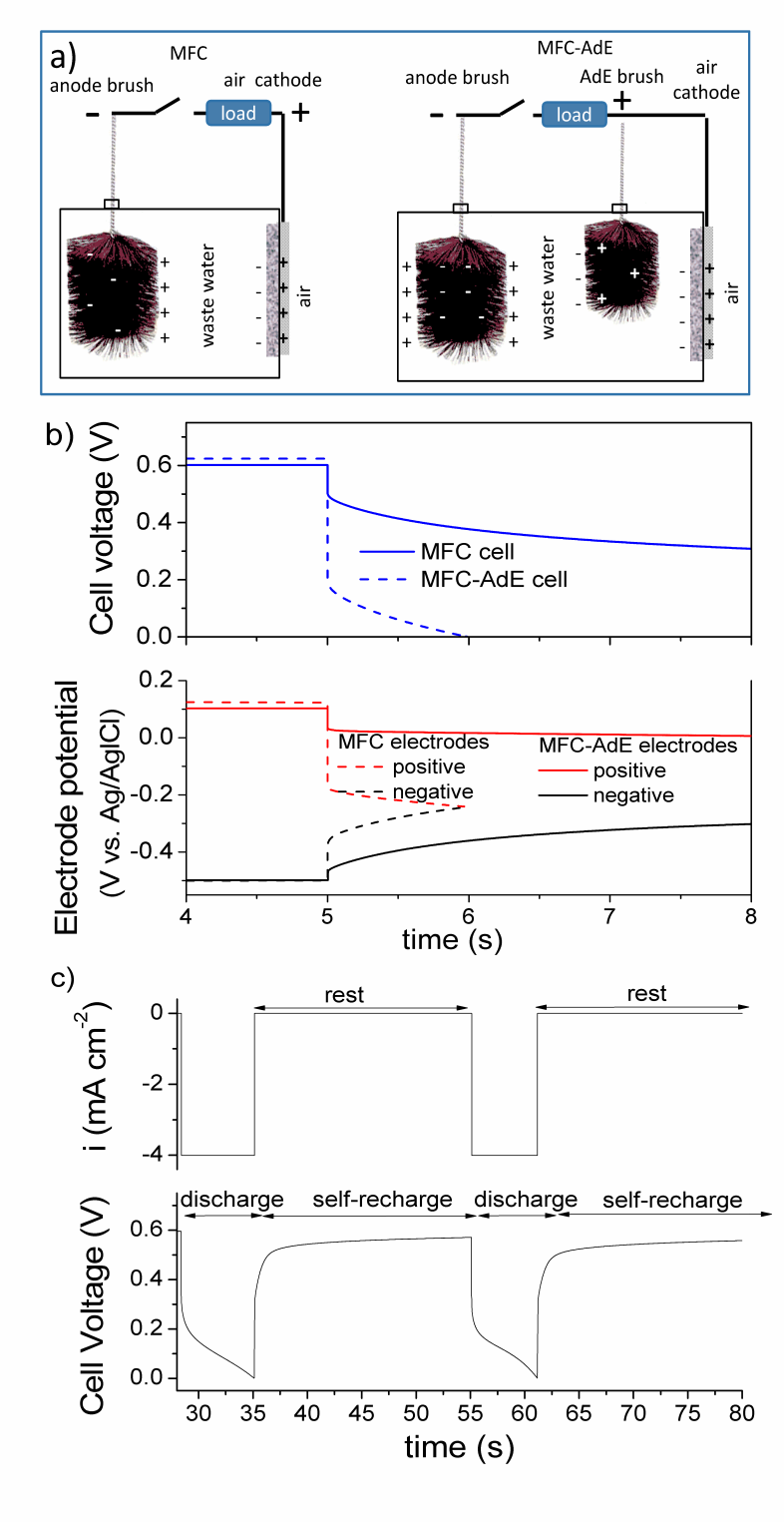


Figure S2. a) Formation of electrochemical double layers at the electrode/waste water interfaces of an MFC and an MFC–AdE where the AdE brush is short circuited with the MFC cathode; b) cell voltage and positive and negative electrode potential profiles of an MFC (dashed lines) and an MFC-AdE (solid lines) under discharge at 2 mA cm^-2^;c) Cell voltage of an MFC-AdE trend during two discharges at 4 mA cm^-2^ each followed by rest.

1. **Supplemental Voltammetric data**

Figure S3. CVs of (a) 200 nm (200-nsC) and (b) 500 nm (500-nsC)-thick electrodes in the IL N_1113_TFSI at different scan rates.

Figure S4. CVs of 200-nsC electrodes in (a) in NaCl 0.01 M and (b) PBS 0.05 M aqueous solutions at different scan rates.

**References**

[1] J. Sayago, X. Meng, F. Quenneville, S. Liang, E. Bourbeau, F. Soavi, F. Cicoira, C. Santato, Electrolyte-gated polymer thin film transistors making use of ionic liquids and ionic liquid-solvent, J. Appl. Phys. 117 (2015) 112809-112814.

[2] X. Meng, F. Quenneville, F. Venne, E. Di Mauro, D. Isık, M. Barbosa, Y. Drolet, Marta M.Natile, F. Soavi, D. Rochefort, C. Santato, Electrolyte-Gated WO_3_ Transistors: Electrochemistry, Structure, and Device Performance, J. Phys. Chem. C, Nanomaterials and Interfaces, 119 (2015) 21732-21738.

[3] V.A. Lanas, Y. Ahn, B.E. Logan, Effects of carbon brush anode size and loading on microbial fuel cell performance in batch and continuous mode, J. Power Sources 247 (2013) 228-234.

[4] C. Santoro, K. Artyushkova, S. Babanova, P. Atanassov, I. Ieropoulos, M. Grattieri, P. Cristiani, S. Trasatti, B. Li, A.J. Schuler, Parameters Characterization and Optimization of Activated Carbon (AC) Cathodes for Microbial Fuel Cell Application, Biores. Technol. 163 (2014) 54-63.

[5] C. Santoro, F. Soavi, A. Serov, C. Arbizzani, P. Atanassov, Self-powered supercapacitive microbial fuel cell: The ultimate way of boosting and harvesting power, Biosens. Bioelectron. 78 (2016) 229-235.

[6] J. Sayago, F. Soavi, Y. Sivalingam, F. Cicoira, C. Santato, Low voltage electrolyte-gated organic transistors making use of high surface area activated carbon gate electrodes, J. Mater. Chem. C 2 (2014) 5690-5694.

[7] J. Sayago, U. Shafique, F. Soavi, F. Cicoira, C. Santato, TransCap: a monolithically integrated supercapacitor and electrolyte-gated transistor, J. Mater. Chem. C, 2 (2014) 10273-10276.

[8] H. Rismani-Yazdi, S.M. Carver, A.D. Christy, O.H. Tuovinen, Cathodic limitations in microbial fuel cells: An overview, J. Power Sources 180 (2008) 683-694.

[9] U. Schröder, Anodic electron transfer mechanisms in microbial fuel cells and their energy efficiency, Phys. Chem. Chem. Phys. 9 (2007) 2619-2629.

[10] F. Béguin, V. Presser, A. Balducci, E. Frackowiak. Carbons and electrolytes for advanced supercapacitors, Adv. Mater. 26 (2014) 2219–2251.
